# Molecular Characteristics of the Endometrium in Polycystic Ovary Syndrome with Insulin Resistance

**DOI:** 10.1007/s43032-026-02069-9

**Published:** 2026-03-16

**Authors:** Yingying Wang, Hongqing Zhang, Yijie Hui, Yajuan Zhang, Hongying Shan

**Affiliations:** Reproductive Medicine Department, The First Affiliated Hospital of Shihezi University, No. 107 HongShan Road, Shihezi, 832000 Xinjiang China

**Keywords:** Polycystic ovary syndrome, Insulin resistance, Endometrial dysfunction, RNA-Seq

## Abstract

Polycystic ovary syndrome (PCOS), one of the most common endocrine and reproductive disorders in women of reproductive age, is frequently complicated by insulin resistance (IR), which affects 50%–80% of PCOS patients and is strongly associated with an increased risk of early pregnancy loss. Evidence suggests that IR may contribute to this risk by impairing endometrial function, a notion supported by observed functional improvements following metformin treatment. While previous studies have provided clues regarding PCOS with IR (PCOS-IR) at the ovarian miRNA and endometrial protein levels, the understanding of the upstream transcriptomic events regulating these pathways remains limited. We performed RNA-Seq on endometrial tissues from PCOS-IR and PCOS without IR (PCOS-NIR), thereby identifying 339 common differentially expressed genes (DEGs) using DESeq2 and Limma. Functional enrichment analysis revealed that DESeq2-identified genes primarily participated in cellular signaling, metabolic regulation, and immune-related pathways. Further weighted gene co-expression network analysis (WGCNA) identified gene modules highly correlated with the PCOS-IR phenotype. Integrating multi-step strategies, including random forest analysis and STRING interaction networks, ultimately identified *FGF17*, *AKT3*, and *IRS4* as key candidate genes. Quantitative real-time PCR (qRT-PCR) confirmed that *FGF17* mRNA expression was significantly downregulated in the endometrium of the PCOS-IR group. This finding suggests that *FGF17* may play a pivotal role in PCOS-IR-related endometrial dysfunction, with promise as a therapeutic target to improve reproductive outcomes in patients. These results provide important theoretical support for subsequent mechanistic exploration and clinical intervention.

**Supplementary Information:**

The online version contains supplementary material available at 10.1007/s43032-026-02069-9.

## Introduction

Polycystic ovary syndrome (PCOS) is one of the most prevalent reproductive endocrine disorders in women of reproductive age. It is primarily characterized by hyperandrogenism, polycystic ovarian morphology, and ovulatory dysfunction. Data indicate a global prevalence of PCOS ranging from 10% to 13% [1], with an infertility risk 15 times higher than in non-PCOS patients [2]. Compared to controls, PCOS patients exhibit significantly reduced clinical pregnancy and live birth rates, alongside markedly increased risks of adverse pregnancy outcomes such as miscarriage, preterm birth, gestational diabetes, and gestational hypertension, severely impacting reproductive health in women of childbearing age [3, 4].

Previous studies have primarily focused on the ovaries in PCOS, suggesting that the leading causes of infertility include abnormal follicular development, ovulatory dysfunction, reduced oocyte quality, and impaired embryonic developmental potential [5]. Although ovulation-inducing drugs can significantly improve issues such as abnormal follicular development and ovulatory dysfunction, PCOS patients still exhibit lower cumulative pregnancy rates and higher miscarriage rates [6]. After adjusting for confounding factors such as age, body mass index (BMI), and embryo quality, the late miscarriage rate following embryo transfer remains significantly higher in PCOS than in non-PCOS [7]. To further exclude embryonic factors, normal embryos were transferred to PCOS, yet their implantation and live birth rates remained lower than those of normal controls [8]. These findings suggest that, beyond oocyte and embryonic factors, abnormal endometrial function may be a key contributor to adverse pregnancy outcomes in PCOS patients.

Existing research suggests that impaired endometrial function in PCOS results from the combined effects of multiple mechanisms, primarily including steroid hormone receptor dysfunction, amino acid metabolism disorders, insulin resistance (IR), activation of inflammatory responses, an imbalance in the immune microenvironment, and abnormal angiogenesis [9–11]. Approximately 50%–80% of PCOS patients exhibit IR [12], which is not only associated with an increased risk of early pregnancy loss [13], but also engages in a vicious cycle with factors including obesity, chronic inflammation, oxidative stress, gut microbiota dysbiosis, and adipose tissue extracellular matrix remodeling [14]. These factors synergistically disrupt endometrial function, leading to pregnancy failure. Notably, after pharmacological interventions improve IR in PCOS, their impaired glucose metabolism is corrected, and concurrently, endometrial function also improves [15, 16]. This suggests that IR may play a central role in the systemic pathological process of PCOS. Given the central role of IR in the pathophysiology of PCOS, it is imperative to conduct an in-depth analysis of its downstream molecular mechanisms. There is a growing shift in PCOS research from systemic metabolism to tissue-specific molecular mechanisms. This evolving focus aims to elucidate the intricate pathways through which IR contributes to reproductive impairment in PCOS. In ovarian tissue, differentially expressed miRNAs may participate in the pathophysiological process of PCOS with IR (PCOS-IR) [17]. Although proteomics has identified endometrial candidate biomarkers associated with PCOS-IR (e.g., ACTR1A and CKB) [18], these findings point to potential effector molecules, the upstream transcriptional regulatory events that cause their differential expression remain unknown, and the transcriptomic molecular characteristics remain unclear.

To investigate this, we compared endometrial transcriptomes from PCOS-IR and PCOS without IR (PCOS-NIR) using RNA sequencing. The aim was to systematically identify distinct transcriptomic features of PCOS-IR, laying the foundation for the discovery of potential biomarkers and therapeutic targets.

## Material and Methods

### Participant and Group Criteria

This study included 15 PCOS patients, divided into PCOS-NIR (*n* = 8) and PCOS-IR (*n* = 7) groups. The inclusion criteria for these patients were: 1) diagnosis based on the 2003 Rotterdam criteria [19]; 2) confirmed PCOS and underwent infertility exploratory surgery or hysteroscopy at The First Affiliated Hospital of Shihezi University; 3) age between 24 and 40 years; 4) postoperative pathological confirmation of proliferative endometrial tissue; 5) IR status determined through a combined assessment using three calculation methods: the IR group required homeostatic model assessment of insulin resistance (HOMA-IR) ≥ 2.69, ratio of fasting insulin to fasting glucose (IGI) > 2.4, and Matsuda insulin sensitivity index (ISI Matsuda) < 4.3; the non-IR group required HOMA-IR < 2.69, IGI ≤ 2.4, and ISI Matsuda ≥ 4.3. Exclusion criteria included: 1) use of hormonal medications within the past 3 months; 2) pregnancy or lactation in the previous 6 months; 3) other conditions such as uterine malformations, adenomyosis, chromosomal abnormalities, endocrine disorders, hyperprolactinemia, thyroid disease, or autoimmune diseases.

### Collection of Clinical Data

Collect patient age, BMI, blood pressure (systolic and diastolic), and measure base hormone levels on days 2-5 of the menstrual cycle, including follicle-stimulating hormone (FSH), luteinizing hormone (LH), prolactin (PRL), estradiol (E2), progesterone (P), testosterone (T), androstenedione (A), and anti-Müllerian hormone (AMH). To determine if patients with PCOS also have IR, an oral glucose tolerance test (OGTT) was performed. Fasting blood glucose and plasma insulin levels were measured, along with blood glucose and plasma insulin levels at 30, 60, and 120 min after oral glucose.

### Endometrial Sample Collection

Endometrial tissue samples were collected from PCOS patients undergoing hysteroscopy for infertility at the Department of Reproductive Medicine, The First Affiliated Hospital of Shihezi University. Within 2 hours of collection, tissue specimens were washed 2–3 times with phosphate-buffered saline (PBS) to remove surface mucus and blood clots. During washing, tissue edges were grasped only with fine forceps to prevent damage. After washing, samples were rapidly frozen in liquid nitrogen and stored at -80 °C. All specimens were confirmed as endometrium in the proliferative phase by two senior pathologists.

### RNA-Seq

RNA extraction and sequencing were performed in collaboration with Shanghai OE Biotechnology Co., Ltd. (Shanghai, China).

### RNA-Seq Data Processing

High-quality clean reads were obtained from the raw FASTQ data through a comprehensive preprocessing workflow. Adapter sequences and low-quality bases were trimmed using Trimmomatic (0.39), and data quality was assessed with Fastp (0.22.0). The high-quality clean reads were then aligned to the human reference genome (GRCh38) using the splice-aware aligner HISAT2 (2.2.1), producing output in the Sequence Alignment/Map (SAM) format. These SAM files were subsequently converted, sorted, and compressed into Binary Alignment/Map (BAM) format using SAMtools (1.6). Gene-level read quantification was performed on the BAM files using featureCounts (2.0.3), during which reads with a mapping quality score below 10 were discarded. Finally, the raw count matrix was filtered to remove genes with no expression in over 50% of the samples and normalized using the Transcripts Per Million (TPM) method for downstream analysis.

### Abnormal Sample Correction

After standardizing mRNA expression levels, all samples underwent cluster analysis and evaluation of gene expression distributions to identify and remove samples with abnormal expression patterns. Subsequently, Principal Component Analysis (PCA) and Partial Least Squares Discriminant Analysis (PLS-DA) were used to assess intra-group reproducibility and inter-group separation trends, thereby evaluating the stability of the experimental system and the validity of grouping. Additionally, Pearson correlation coefficients quantified the similarity of expression profiles between samples. Visualization of the results was performed using the R package ggplot2 (4.2.1).

### Identification of DEGs

Using DESeq2 (1.36.0) and Limma (3.52.2), we identified differentially expressed genes (DEGs) between the PCOS-NIR and PCOS-IR groups. The screening threshold was set at *P* < 0.05 and |log₂(Fold Change)|≥ 1. The final results were visualized with ggplot2 (4.2.1) and ComplexHeatmap (2.13.1).

### GO and KEGG Enrichment Analysis of DEGs

To clarify the biological processes and signaling pathways involved in PCOS-IR-related DEGs, this study used the online database, the Database for Annotation, Visualization, and Integrated Discovery (DAVID), to perform Gene Ontology (GO) functional enrichment analysis and Kyoto Encyclopedia of Genes and Genomes (KEGG) pathway enrichment analysis. A significance threshold of *P* < 0.05 was set to identify statistically significant GO terms and KEGG pathways. Enrichment analysis results were visualized using the R package ggplot2 (4.2.1).

### Weighted Gene Co-expression Network Analysis (WGCNA)

This study used WGCNA to identify co-expressed gene modules linked to PCOS and IR phenotypes, employing the R package WGCNA (1.1.25). We applied WGCNA to find modules associated with these conditions. First, raw RNA-Seq count data were preprocessed to remove genes with low expression variability (standard deviation ≤ 0.5). Then, based on the biological principle of scale-free networks, we determined the optimal soft threshold for calculating the adjacency matrix. The dynamic tree cut algorithm was used to identify gene modules with similar expression patterns. A weighted co-expression network was constructed, grouping genes into modules based on their expression profiles. To visualize the relationships among modules, heatmaps were generated to display correlations among module-specific genes. Finally, Pearson correlation analysis was conducted to assess the association between each module's key genes and clinical traits, using correlation coefficients and *p*-values to select significant modules.

### Screening of Key Genes

This study employed a multi-step integrated approach to identify key genes linked to PCOS-IR. First, DEGs identified through DESeq2 and Limma differential analysis were combined with genes from WGCNA key modules and known IR-related genes (Table 2) for integrated analysis. A Venn diagram was created using the R package ggvenn (0.1.9) to visualize overlapping genes. Then, using the R package randomForest (4.7.1.1), the weights for each gene were estimated via random forests, and the results were visualized with ggplot2 (3.3.6). Finally, the Friends analysis was performed. The gene interaction network was constructed using the R package GOSemSim (2.22.0), and the gene significance was evaluated based on network topological metrics. The results were presented as a cloud-rain diagram using ggplot2 (4.2.1).

### Quantitative Real-time PCR (qRT-PCR) Analysis

QRT-PCR was used to measure mRNA levels of *FGF17*, *AKT3*, and *IRS4* in endometrial tissues from 7 PCOS-NIR patients and 7 PCOS-IR patients. The reaction system had a total volume of 20 μL. Using the SYBR Green qPCR kit, a mixture containing 10 μL of real-time PCR mix, 1 μL of upstream primer, 1 μL of downstream primer, 7 μL of DEPC-treated water, and 1 μL of cDNA template was prepared on ice and added to a 96-well plate for amplification. The reaction program was set as follows: pre-denaturation at 50 °C for 2 minutes (min) and 95 °C for 2 min; 40 cycles of PCR (95 °C for 15 seconds (s), 60 °C for 40 s); melting curve analysis at 95 °C for 15 s, 60 °C for 1 min, and 95 °C for 1 s. Gene expression levels were calculated using the 2^(-ΔΔCT) method, with 18S rRNA serving as the internal reference gene. The primers used for qRT-PCR are listed in Supplementary Table 1.

### Statistical Analysis

Statistical analyses were performed using R (4.2.2), SPSS (26.0), and GraphPad Prism (10). Normality was assessed with the Shapiro–Wilk test, Kolmogorov–Smirnov test, or D'Agostino-Pearson omnibus K^2^ test. Normally distributed quantitative data are reported as mean ± standard deviation and compared between groups with t-tests. Non-normally distributed data are presented as median and interquartile range (25%–75%), with group comparisons conducted using nonparametric rank-sum tests. Correlations between samples, gene modules, and phenotypes were evaluated using Pearson correlation coefficients. *P*-value < 0.05 was considered statistically significant.

## Results

### Clinical Characteristics

Compared with the PCOS-NIR group, the PCOS-IR group showed significantly higher body weight, BMI, and IR-related indicators (INS 0, INS 30, INS 60, INS 120, HOMA-IR, IGR, and ISI Matsuda) than the control group. However, there were no significant differences in age, FSH, LH, E2, T, GLU 0, GLU 30, GLU 60, and GLU 120 between the two groups (Table 1).

**Table 1:** Characteristics of PCOS-IR and PCOS-NIR

Characteristic	PCOS-NIR(*N* = 8)	PCOS-IR(*N* = 7)	X2/Z/t	*p*-value
Age (year)	28.50 ± 2.45	27.71 ± 2.81	0.335	0.573
Weight (kg)	54.75 ± 9.18	70.00 ± 12.11	7.681	0.016
BMI (kg/m^2^)	21.98 ± 3.21	26.08 ± 3.43	5.746	0.032
FSH (U/L)	5.60 ± 1.40	5.54 ± 1.31	0.006	0.938
LH (U/L)	7.67 ± 4.33	7.10 ± 3.93	0.072	0.793
E2 (pmol/L)	144.00 (111.06–188.88)	150.83 (130.50–178.50)	0.121	0.728
T (μg/L)	0.75 (0.70–0.82)	0.69 (0.69–0.82)	0.413	0.521
GLU 0 (mmol/L)	4.67 ± 0.33	5.07 ± 0.58	2.754	0.121
GLU 30 (mmol/L)	7.35 ± 1.22	8.01 ± 1.84	0.696	0.419
GLU 60 (mmol/L)	6.64 ± 0.64	7.69 ± 2.12	1.788	0.204
GLU 120 (mmol/L)	5.90 ± 1.21	6.26 ± 1.39	0.283	0.603
INS 0 (μU/mL)	7.10 ± 1.77	18.21 ± 3.49	63.207	< 0.001
INS 30 (μU/mL)	50.91 (36.82–71.74)	98.43 (80.96–113.33)	4.835	0.028
INS 60 (μU/mL)	47.48 ± 17.18	124.81 ± 39.92	24.963	< 0.001
INS 120 (μU/mL)	35.31 (33.05–48.06)	74.16 (50.79–97.23)	6.482	0.011
HOMA-IR	1.48 ± 0.39	3.98 ± 0.91	50.177	< 0.001
IGR	1.52 ± 0.38	3.73 ± 0.74	54.717	< 0.001
ISI Matsuda	6.91 ± 1.92	2.61 ± 0.61	32.129	< 0.001

*PCOS* Polycystic ovary syndrome; *BMI* Body mass index; *FSH* Follicle-stimulating hormone; *LH* Luteinizing hormone; *E2* Estradiol; *T* Testosterone; *GLU0* Fasting blood glucose; *GLU30* Blood glucose 30 min after oral glucose; *GLU60* Blood glucose 60 min after oral glucose; *GLU120* Blood glucose 120 min after oral glucose; *INS0* Fasting insulin; *INS30* Insulin 30 min after oral glucose; *INS60* Insulin 60 min after oral glucose; *INS120* Insulin 120 min after oral glucose; *HOMA-IR* Homeostatic model assessment of insulin resistance; *IGR *Ratio of fasting insulin to fasting glucose; *ISI Matsuda *Matsuda insulin sensitivity index

### RNA-Seq Sequencing Data Quality Control

**Fig. 1:**
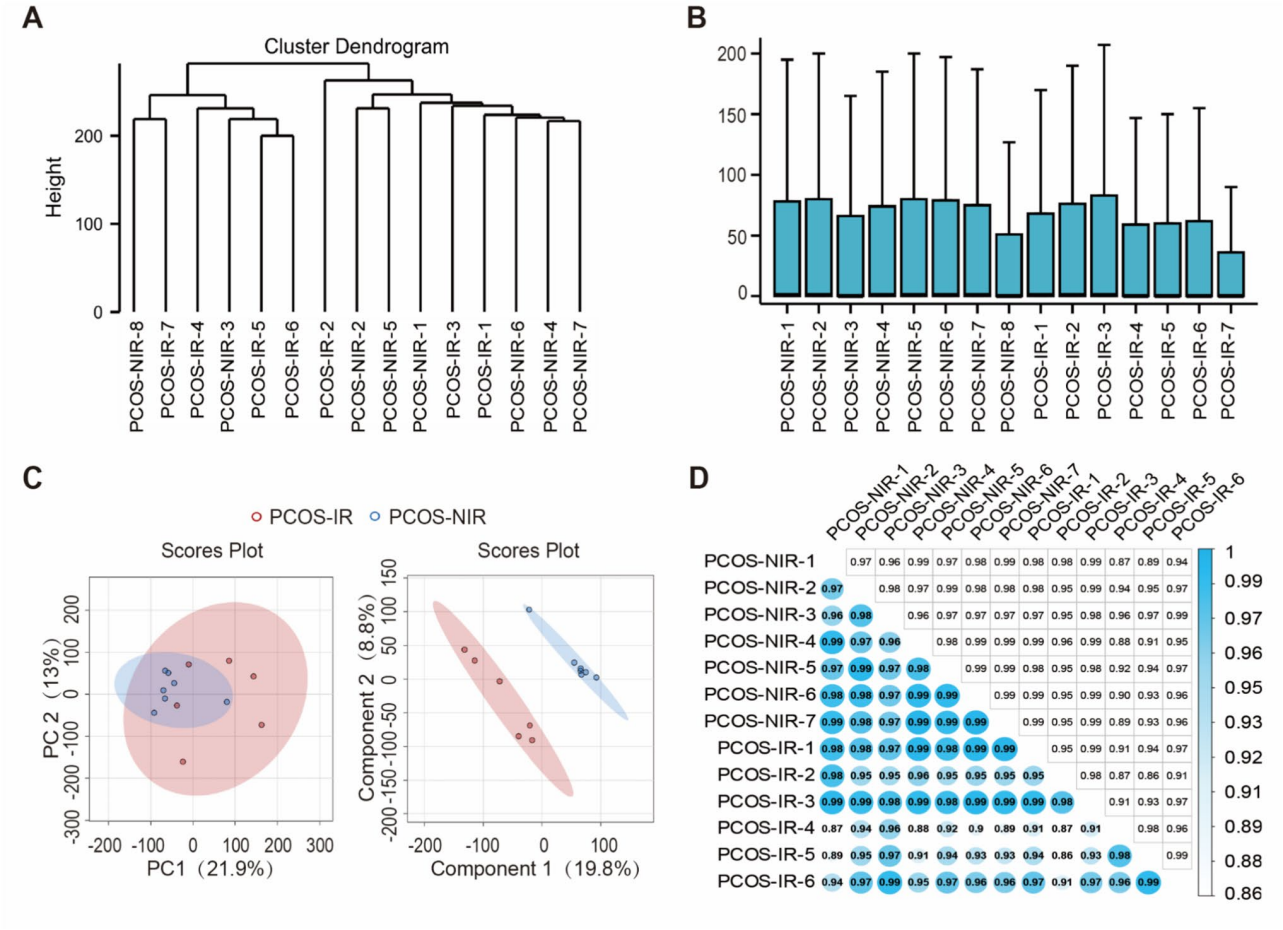
Quality control of RNA-Seq data from two groups of endometrial tissue. **A**, Clustering dendrogram of proliferative phase endometrial RNA-Seq samples in the PCOS-NIR group and PCOS-IR group (PCOS-NIR group *n* = 8, PCOS-IR group *n* = 7); **B**, Box plots of gene expression levels from proliferative phase endometrial RNA-Seq sequencing in the PCOS-NIR group and PCOS-IR group (PCOS-NIR group *n* = 8, PCOS-IR group *n* = 7); **C**, PCA and PLS-DA analysis of proliferative phase endometrial RNA-Seq samples from PCOS-NIR and PCOS-IR groups (PCOS-NIR group *n* = 7, PCOS-IR group *n* = 6); **D**, Heatmap of correlation analysis for proliferative phase endometrial RNA-Seq samples in PCOS-NIR and PCOS-IR groups (PCOS-NIR group *n* = 7, PCOS-IR group *n* = 6). For **D**, data was analyzed using the Pearson correlation algorithm

To assess the quality of RNA-Seq data from two groups of endometrial tissue samples, we conducted systematic quality control analyses. First, the sample clustering dendrogram revealed that all samples grouped into two clusters with no clear outliers (Fig. 1A). Boxplot analysis indicated that PCOS-NIR-8 and PCOS-IR-7 samples had significantly lower expression levels than other samples (Fig. 1B), leading to their removal from further analyses. Second, PCA was used to reduce the dimensionality of the endometrial RNA-Seq gene expression data from both groups. PCA showed no distinct separation between the groups, nor was intra-group clustering significant. This may be because both groups originate from the PCOS disease cohort. Therefore, PLS-DA was further employed for supervised pattern recognition. The PLS-DA results demonstrated clear separation between groups and distinct intra-group clustering, with non-overlapping 95% confidence ellipses, indicating good reproducibility within groups and significant differences between them (Fig. 1C). Quality assessment based on Pearson correlation revealed strong positive correlations among all samples (r > 0.8) (Fig. 1D). In summary, the sequencing data quality is reliable and meets the standards required for subsequent transcriptome differential expression analysis.

### Identification and Functional Analysis of Differentially Expressed Genes

**Fig. 2:**
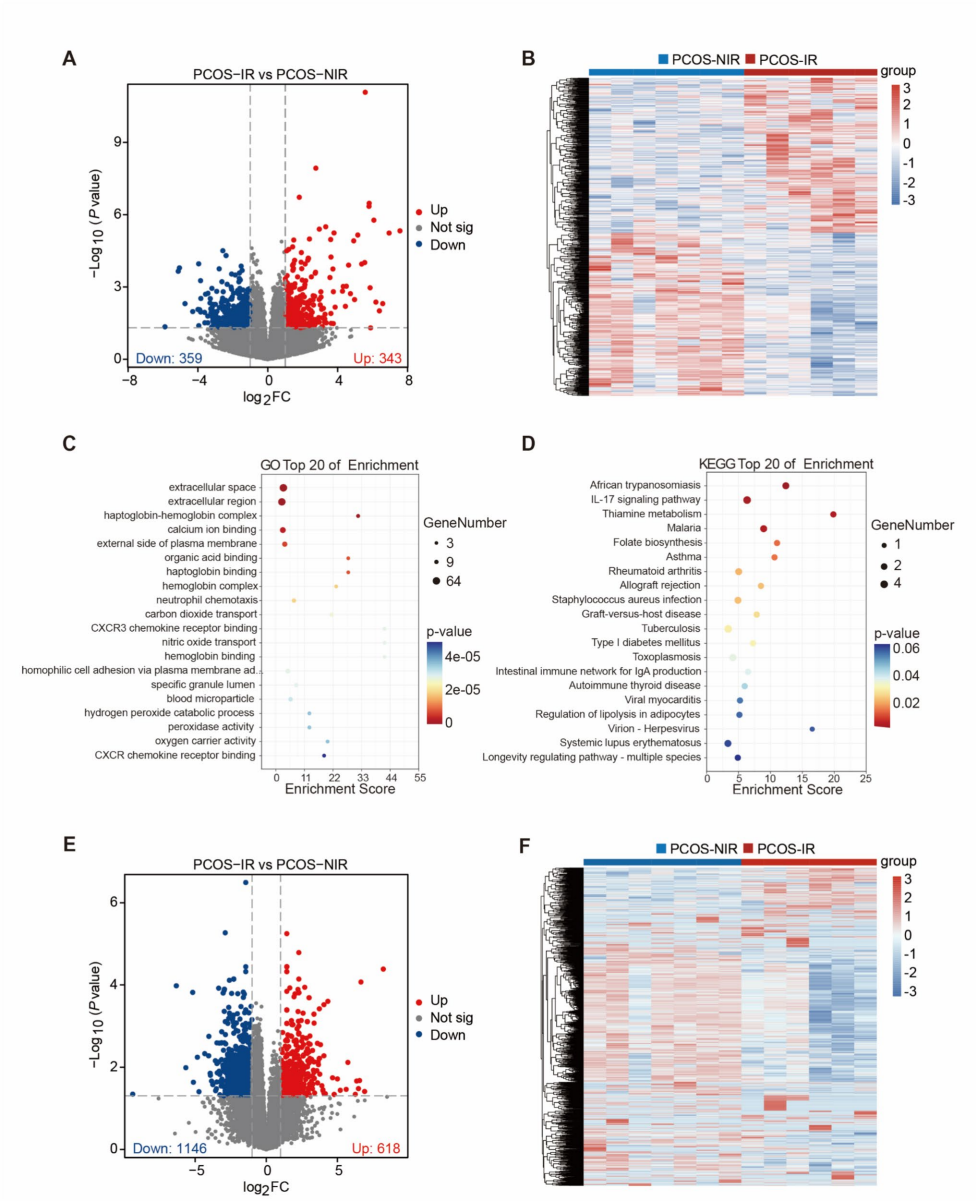
Differential expression analysis and functional enrichment analysis of endometrial tissue in two groups. **A**, Volcano plot of DESeq2-identified DEGs from proliferative phase endometrial tissue RNA-Seq analysis in PCOS-NIR and PCOS-IR groups; **B**, Heatmap of DESeq2-identified DEGs from proliferative phase endometrial tissue RNA-Seq analysis in PCOS-NIR and PCOS-IR groups; **C**, GO enrichment analysis of DEGs from proliferative phase endometrial tissue RNA-Seq analysis in PCOS-NIR and PCOS-IR groups; **D**, KEGG enrichment analysis of DEGs from proliferative phase endometrial tissue RNA-Seq analysis in PCOS-NIR and PCOS-IR groups; **E**, Volcano plot of Limma-identified DEGs from proliferative phase endometrial tissue RNA-Seq analysis in PCOS-NIR and PCOS-IR groups; **F**, Heatmap of Limma-identified DEGs from proliferative phase endometrial tissue RNA-Seq analysis in PCOS-NIR and PCOS-IR groups. (*P* < 0.05,|log_2_FC|≥ 1)

To examine differences in endometrial tissue RNA-Seq data between the PCOS-NIR and PCOS-IR groups, differential analysis and visualization were conducted. DESeq2 identified 702 DEGs, including 343 significantly upregulated and 359 significantly downregulated genes. The results were visualized using volcano plots and heatmaps (Fig. 2A-B). To explore the functional relevance of these DEGs, enrichment analysis was performed. GO enrichment analysis showed that DEGs from both groups were significantly enriched in the “extracellular space” and “extracellular region”, indicating the presence of active intercellular communication in PCOS-IR (Fig. 2C). KEGG enrichment analysis revealed that DEGs in both groups were significantly involved in pathways related to the immune system, such as “systemic lupus erythematosus”, “graft-versus-host disease”, and the "IL-17 signaling pathway”. Additionally, pathways associated with the endocrine and metabolic systems were also notably enriched, including “type 1 diabetes mellitus” and “regulation of adipocyte lipolysis” (Fig. 2D). To improve the quality of DEGs screening, the Limma package was used for re-analysis. Limma identified 1,764 DEGs, with 618 significantly upregulated and 1,146 significantly downregulated genes. These results were visualized with volcano plots and heatmaps (Fig. 2E-F).

### Construction of Weighted Gene Co-expression Networks and Identification of Key Modules

**Fig. 3:**
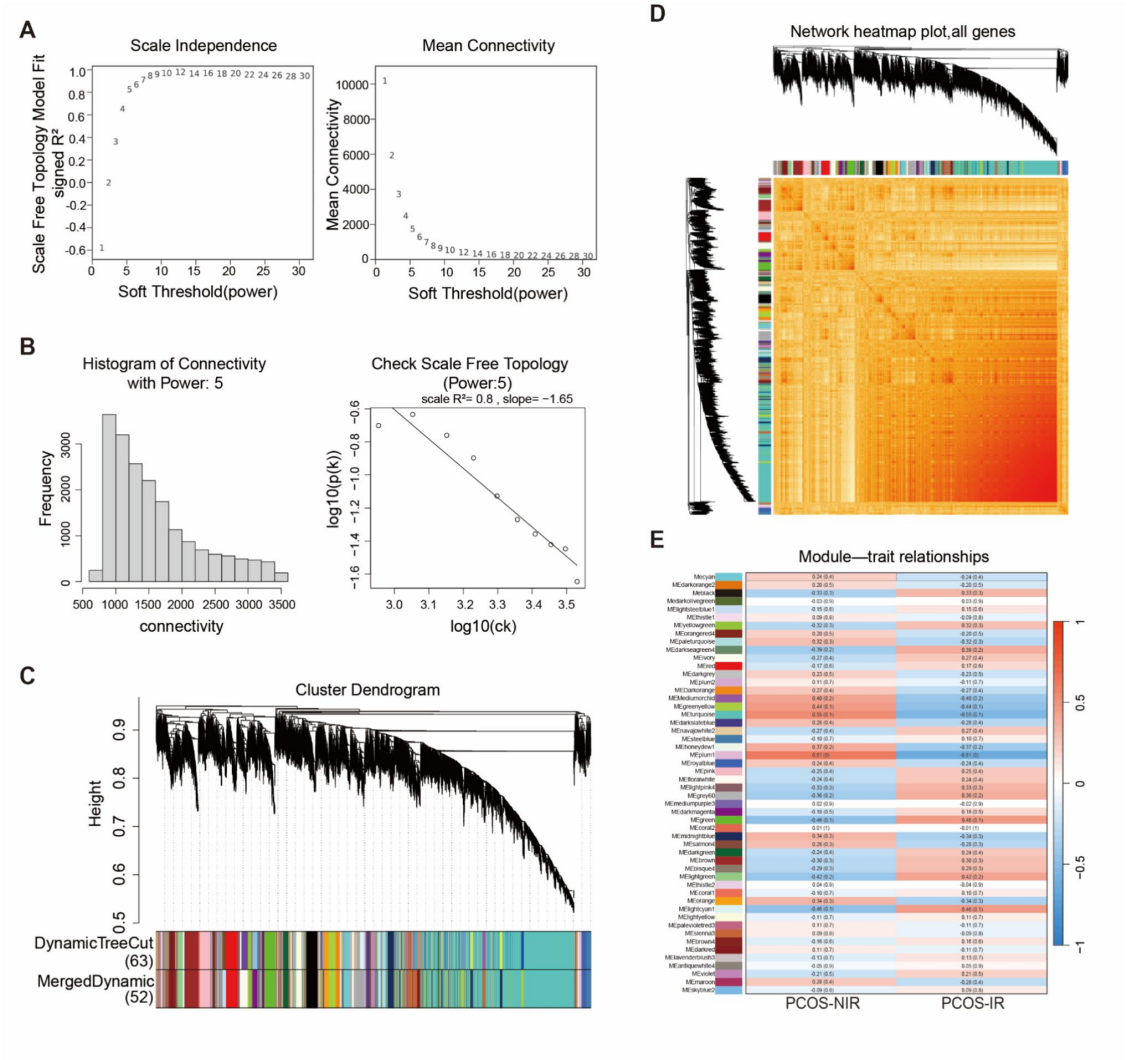
Weighted co-expression network construction and identification of key modules. **A**, Soft thresholding construction: Soft threshold determined based on a scaling topologies fit index (R^2^) of 0.8 for network construction; **B**, Average connectivity analysis: Average connectivity of genes at soft threshold (power) = 5; **C**, Gene clustering dendrogram and module color identification: Gene clustering dendrogram based on the topological overlap matrix; **D**, Module signature gene clustering heatmap; **E**, Module-trait association heatmap: Displays Pearson correlation coefficients between each gene module and clinical features

To identify co-expressed gene modules linked to PCOS-IR clinical features, this study performed WGCNA on RNA-Seq data from two groups of endometrial tissues. First, network construction parameters were set using soft thresholding. When the soft threshold (power) was set to 5, the scale-free topology fit index R^2^ reached 0.8 (Fig. 3A-B), indicating the network displayed near-scale-free characteristics. This parameter was therefore used for the remaining analysis. Based on this, a weighted gene co-expression network was built, clustering genes into 52 modules. The gray module represented unclassified genes and was excluded from further analysis (Fig. 3C). The module clustering heatmap showed the expression patterns and structural relationships of genes within each module (Fig. 3D). Pearson correlation analysis was then used to evaluate the association between module-specific genes and PCOS-IR traits. Results showed the highest correlation between the MEplum1 module and the IR phenotype (correlation coefficient = 0.61, *P* < 0.05), indicating that MEplum1's genes have the strongest association with PCOS-IR clinical features (Fig. 3E). Consequently, MEplum1 was selected as the key module for further analysis.

### Screen for Key Genes

**Fig. 4:**
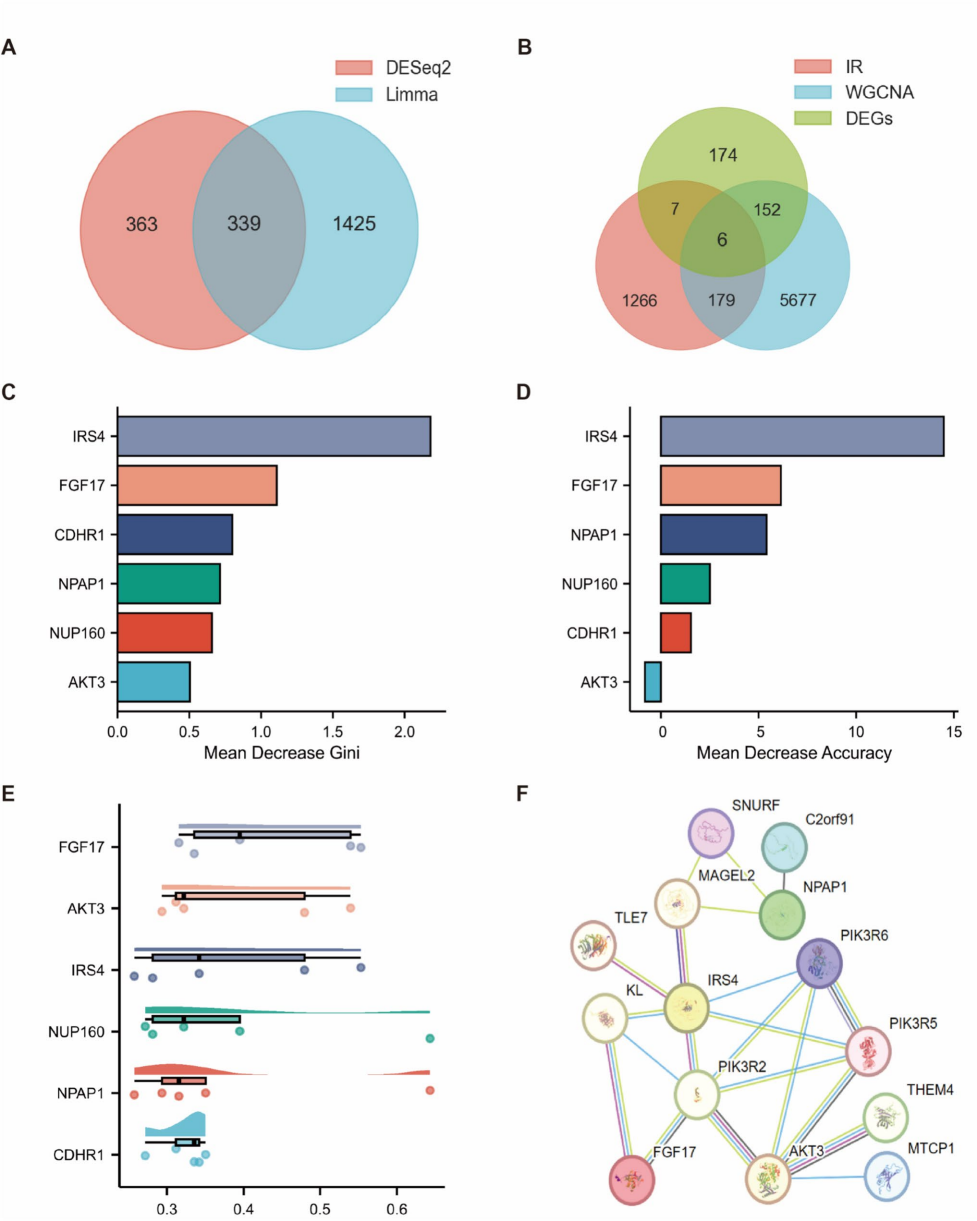
Screening of two groups of key genes. **A**, Venn diagram of DEGs identified by DESeq2 and Limma algorithms in proliferative phase endometrial tissue from PCOS-NIR and PCOS-IR groups; **B**, Venn diagram of proliferative phase endometrial tissue DEGs, IR-related genes, and WGCNA key modules in PCOS-NIR and PCOS-IR groups; **C**, Random forest algorithm mean decrease Gini; **D**, Random forest algorithm mean decrease accuracy; **E**, Friends analysis cloud-rain plot; **F**, STRING interaction network diagram

To identify key genes in endometrial tissue RNA-Seq data between the PCOS-NIR and PCOS-IR groups, subsequent analyses included random forests, Friends analysis, and STRING network analysis. First, the DESeq2 and Limma algorithms were used to identify DEGs in the RNA-Seq data from both groups' endometrial tissues, and the results were intersected and visualized. The Venn diagram revealed 339 overlapping DEGs between the two groups (Fig. 4A). Next, 1,458 IR-related genes were retrieved from the MSigDB database and GeneCard website (Table 2). An integrated analysis was performed by combining the DEGs identified by both DESeq2 and Limma, IR-related genes, and the MEplum1 module from WGCNA, followed by visualization. The Venn diagram identified six key DEGs (Fig. 4B). These six DEGs were then analyzed using the Random Forest algorithm to assess their Mean Decrease Gini and Mean Decrease Accuracy. The genes were ranked based on these features (Fig. 4C-D). Subsequently, the friends analysis was performed on the six key DEGs. The cloud-rain diagram illustrated the similarity between each gene and others, revealing that *FGF17*, *AKT3*, and *IRS4* had the most significant similarity with other genes, confirming them as the final key genes (Fig. 4E). Finally, STRING network analysis showed that FGF17, AKT3, and IRS4 all interact with PIK3R2 (Fig. 4F). These results suggest that *FGF17*, *AKT3*, and *IRS4* act as crucial hub genes between endometrial tissues from the PCOS-NIR and PCOS-IR groups, potentially interacting with PIK3R2 to influence PCOS-IR-related biological processes.

**Table 2:** Insulin resistance - related genes

	Standard name	Data source	Number of genes
1	BIOCARTA_INSULIN_PATHWAY	MSigDB	27
2	HP_INSULIN_RESISTANCE	MSigDB	107
3	HP_INSULIN_RESISTANT_DIABETES_MELLITUS	MSigDB	39
4	PID_INSULIN_PATHWAY	MSigDB	44
5	WP_INSULIN_SIGNALING	MSigDB	160
6	REACTOME_REGULATION_OF_INSULIN_SECRETION	MSigDB	78
7	REACTOME_SIGNALING_BY_INSULIN_RECEPTOR	MSigDB	82
8	KEGG_INSULIN_SIGNALING_PATHWAY	MSigDB	137
9	HP_IMPAIRED_GLUCOSE_TOLERANCE	MSigDB	37
10	HP_INCREASED_C_PEPTIDE_LEVEL	MSigDB	14
11	HP_ABNORMAL_CIRCULATING_INSULIN_CONCENTRATION	MSigDB	156
12	HP_ABNORMAL_ORAL_GLUCOSE_TOLERANCE	MSigDB	15
13	HP_ABNORMAL_RESPONSE_TO_GLUCAGON_STIMULATION_TEST	MSigDB	5
14	HP_ABNORMAL_RESPONSE_TO_INSULIN_TOLERANCE_TEST	MSigDB	8
15	GOMF_INSULIN_LIKE_GROWTH_FACTOR_BINDING	MSigDB	19
16	GOMF_INSULIN_RECEPTOR_BINDING	MSigDB	22
17	GOMF_INSULIN_RECEPTOR_SUBSTRATE_BINDING	MSigDB	13
18	GOBP_REGULATION_OF_INSULIN_RECEPTOR_SIGNALING_PATHWAY	MSigDB	73
19	GOBP_RESPONSE_TO_INSULIN	MSigDB	277
20	GOBP_INSULIN_RECEPTOR_SIGNALING_PATHWAY	MSigDB	131
21	GOBP_CELLULAR_RESPONSE_TO_INSULIN_STIMULUS	MSigDB	214
22	BIOCARTA_LEPTIN_PATHWAY	MSigDB	11
23	Gene Card (IR) Relevance score ≥ 10	Gene Card	945

### Validation of Key Genes

**Fig. 5:**
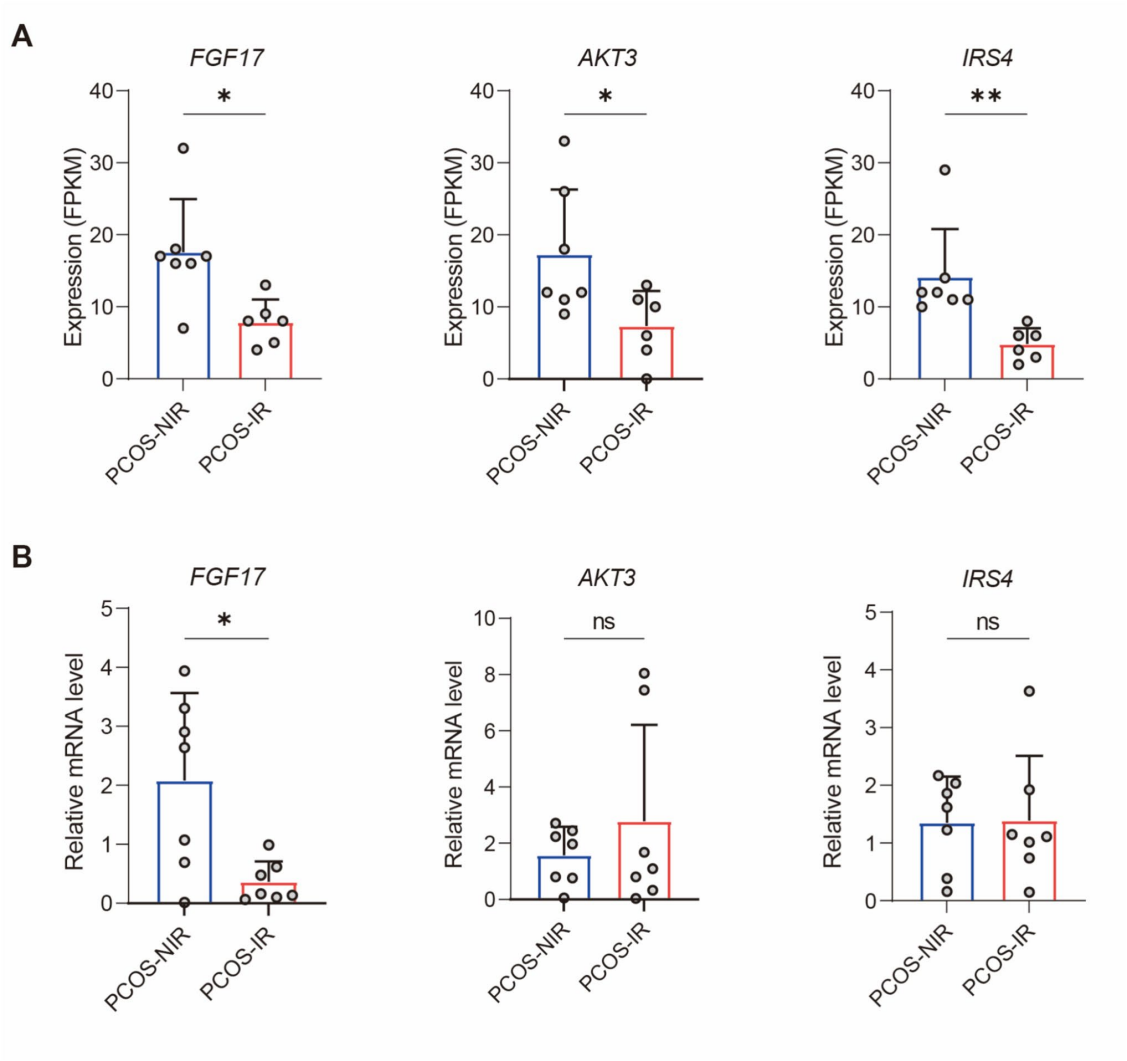
Expression of key genes in proliferative phase endometrial tissue from both groups. **A**, Average expression levels of key genes *FGF17*, *AKT3*, and *IRS4* in proliferative phase endometrial tissue RNA-Seq data from the PCOS-NIR group and PCOS-IR group (PCOS-NIR group *n* = 7, PCOS-IR group *n* = 6); **B**, Relative mRNA expression levels of key genes *FGF17*, *AKT3*, and *IRS4* in proliferative phase endometrial tissue from PCOS-NIR and PCOS-IR groups, analyzed by qRT-PCR (n = 7 each group). For **A** and **B**, Statistical analysis: Normality was assessed using the D'Agostino-Pearson omnibus test, followed by unpaired t-tests. *P < 0.05, **P < 0.01, ns: no significance

Next, we validated the selected key genes *FGF17*, *AKT3*, and *IRS4*. First, we verified the RNA-Seq data for these genes. Results showed that, compared to the PCOS-NIR group, the PCOS-IR group had significantly lower expression of all three key genes (Fig. 5A). Second, qRT-PCR was used to measure mRNA levels of these genes. Findings indicated that, relative to the PCOS-NIR group, *FGF17* mRNA expression was notably decreased in the endometrial tissue of PCOS-IR patients, consistent with the RNA-Seq results. However, no significant differences were observed in the expression of *AKT3* and *IRS4* (Fig. 5B). These results suggest that altered *FGF17* expression in proliferative-phase endometrial tissue of PCOS-IR patients may serve as a potential therapeutic target for improving endometrial dysfunction in this group.

## Discussion

PCOS is a severe endocrine and metabolic disorder that afflicts women of reproductive age, with a strikingly high prevalence of IR reaching 50% to 80% [12, 20]. This IR is strongly linked to a significantly elevated risk of early pregnancy loss [13]. Research suggests IR likely mediates this adverse outcome by impairing endometrial function, while oral metformin improves endometrial function following IR correction [16]. This highlights the pivotal role of IR in impairing endometrial function in PCOS patients. While previous studies have provided clues regarding PCOS-IR at the ovarian miRNA and endometrial protein levels [17, 18], the understanding of the upstream transcriptional events regulating these pathways remains limited. Therefore, elucidating the molecular characteristics of the PCOS-IR endometrium at the transcriptomic level is crucial.

To systematically elucidate the molecular characteristics underlying PCOS-IR, this study employed a multi-step bioinformatics approach. First, to enhance the reliability of DEGs, we identified 339 common DEGs between the PCOS-IR and PCOS-NIR groups using both DESeq2 and Limma, based on RNA-Seq data. Enrichment analysis of the DESeq2-identified DEGs revealed significant pathway enrichment in the immune system and endocrine metabolism systems (Fig. 2D), consistent with the pronounced chronic immune inflammation and lipid dysregulation observed in PCOS-IR patients [14]. Further WGCNA co-expression network analysis identified MEplum1 as the core module most strongly correlated with the IR phenotype. Integrating common DEGs, MEplum1 module genes, and known IR-associated genes yielded six candidate genes. Finally, by combining random forest algorithms, friends analysis, and STRING interaction networks for in-depth screening, it was revealed that *FGF17*, *AKT3*, and *IRS4* all interact with *PIK3R2*. Thus, *FGF17*, *AKT3*, and *IRS4* were identified as key genes in PCOS-IR and validated via qRT-PCR.

Fibroblast Growth Factor 17 (FGF17), as a member of the FGF family, exhibits abnormal expression significantly associated with various diseases, including leukemia, Parkinson's disease, and pancreatic cancer [21–23]. As a key regulator of oocyte maturation and embryonic development [24, 25], FGF17's role in modulating endometrial function in PCOS patients may be complex. This study observed downregulated *FGF17* mRNA expression in endometrial tissue from PCOS-IR patients, contrasting with previously reported elevated plasma protein levels [26]. This suggests FGF17 may undergo unique post-transcriptional regulatory mechanisms or exhibit distinct paracrine/autocrine action patterns within the PCOS-IR endometrium. Furthermore, multiple in vitro experiments demonstrate that FGF17 inhibits granulosa cell function. For instance, it synergistically reduces FSH-stimulated estradiol and progesterone secretion with FGF18 and collaborates with other FGF family members to disrupt the FSH/IGF1 signaling pathway, thereby impairing dominant follicle development [27]. Combined with the downregulation of FGF17 expression in the endometrium observed in this study, we hypothesize that FGF17 may act through distinct mechanisms in the ovary and uterus, respectively, to contribute to the pathophysiology of PCOS-IR jointly: at the ovarian level, abnormal FGF17 levels may disrupt follicular development and hormonal balance; at the uterine level, its reduced expression may contribute to endometrial dysfunction in PCOS-IR patients by disrupting glycolipid metabolism pathways and local insulin signaling. 

AKT3, as a key member of the AKT protein kinase family, not only participates in regulating the insulin/PI3K-AKT signaling pathway [28], but also modulates endometrial decidualization and the development of uterine fibroids [29, 30]. Furthermore, in reproductive system diseases, AKT3 serves as a critical biomarker for predicting the occurrence of cervical and ovarian cancers [31, 32]. IRS4, a vital member of the insulin receptor substrate (IRS) family, functions as an insulin and IGF-1 inducible protein. Its role extends beyond maintaining systemic glucose homeostasis and central energy balance [33, 34], to critically negatively regulating the IGF-1 signaling pathway by inhibiting IRS1 and IRS2, thereby playing a pivotal role in insulin receptor-related mechanisms [35, 36]. Additional studies indicate reduced IRS4 expression in theca cells of PCOS patients, with its cell-specific downregulation potentially contributing to the regulation of local ovarian hyperandrogenism and the pathological process of theca cell hyperplasia [37]. RNA-Seq in this study revealed downregulation of AKT3 and IRS4 in PCOS-IR endometrium, though qRT-PCR validation showed no significant differences. This discrepancy may stem from variations in sensitivity and specificity between transcriptomic sequencing and validation methods, limited sample size, or indicate complex post-transcriptional regulation of AKT3 and IRS4. Given AKT3's central role in the PI3K-AKT signaling pathway and IRS4's negative regulatory function in insulin signaling, we hypothesize that their co-downregulation in PCOS-IR endometrium may jointly contribute to local insulin signaling pathway or local insulin signaling dysfunction. This dysfunction may represent a critical link in triggering endometrial insulin sensitivity disorders and subsequent metabolic and functional abnormalities. 

Through STRING interaction network analysis, this study identified potential interactions between *FGF17*, *AKT3*, and *IRS4* with *PIK3R2*. AKT3 and PIK3R2, as key regulators of the PI3K-AKT pathway, play crucial roles in angiogenesis and development. Mutations in these genes can cause developmental disorders such as megalencephaly syndromes [38, 39], and are implicated in various diseases, including primary gastric vasculitis, esophageal squamous cell carcinoma, and hypoglycemia due to insulin deficiency [40–42]. In ovarian cancer cells, FER-mediated phosphorylation of IRS4 promotes the recruitment of PIK3R2/p85β, thereby activating the PI3K-AKT signaling pathway [43]. Furthermore, both IRS4 and PIK3R2 are predicted to be highly disordered proteins, and they exhibit synergistic effects in the pathogenesis of type 2 diabetes [44]. Although no direct interaction between FGF17 and PIK3R2 has been documented, both molecules have been reported to participate in PI3K-AKT pathway activation through distinct mechanisms [45, 46]. Notably, KEGG pathway enrichment analysis in this study indicates that *FGF17* and *AKT3* co-enrich in the hsa05218 Melanoma pathway. They may contribute to melanoma development and progression through mechanisms such as influencing key driver gene mutations (e.g., *BRAF*, *CDKN2A*, *NRAS*, and *TP53*), signaling pathway abnormalities (e.g., MAPK/PI3K-AKT activation), and immune microenvironment regulation (e.g., PD-L1 upregulation) in melanoma initiation and progression [47–49]. Therefore, we hypothesize that FGF17 and AKT3 may exhibit functional synergism and, through interaction with PIK3R2, jointly mediate abnormal activation of the PI3K-AKT signaling pathway. Complex interactions among FGF17, AKT3, IRS4, and PIK3R2 within the PI3K-AKT signaling network suggest their potential critical roles in the pathophysiology of PCOS-IR.

In summary, this study employed RNA-Seq to analyze the transcriptomes of endometrial tissues from PCOS-IR and PCOS-NIR patients, establishing a preliminary differential gene expression profile. Bioinformatics analysis indicated that genes such as *FGF17*, *AKT3*, *IRS4*, and *PIK3R2* may regulate metabolic and functional abnormalities in the endometrium of PCOS-IR patients by modulating insulin-related signaling pathways, such as PI3K-AKT. QRT-PCR validation confirmed significantly downregulated *FGF17* expression in the PCOS-IR group. *FGF17* may serve not only as a potential biomarker for PCOS-IR but also as a therapeutic target. However, this study has certain limitations. Although we proposed a potential regulatory network based on multi-gene interactions, the existing literature supporting direct associations between these genes—particularly *FGF17*, *AKT3*, and *IRS4*—and PCOS-IR remains relatively limited. Furthermore, the small sample size may limit the generalizability and statistical power of the findings, and it is challenging to fully capture the heterogeneity of the PCOS patient population, including those with or without IR. Nevertheless, this study provides new clues and a theoretical basis for the molecular mechanisms of PCOS-IR. Based on current findings, future work should expand sample sizes and validate gene expression and mechanisms using protein-level and functional experiments (e.g., in vitro knockdown/overexpression and animal models). This will further elucidate the specific mechanisms and regulatory networks underlying genes such as *FGF17*, *AKT3*, and *IRS4* in the PCOS-IR endometrium. By systematically elucidating these genes and their pathways, we may uncover novel mechanistic insights and potential therapeutic targets for PCOS-IR endometrial pathology. Ultimately, this research aims to improve endometrial function in PCOS patients, enhance reproductive outcomes, and reduce long-term metabolic and perinatal complications.

## Supplementary Information

Below is the link to the electronic supplementary material.

Supplementary file1 (DOCX 17.6 KB)

## Data Availability

The sequencing data generated in this study are publicly available in the Genome Sequence Archive (GSA) for Human under accession number HRA013762. Source data are provided with this paper.
