# Supplementary material for: Molecular Characteristics of the Endometrium in Polycystic Ovary Syndrome with Insulin Resistance
Source: Reprod Sci. 2026 Mar 16;33(3):643–56. doi: 10.1007/s43032-026-02069-9 (PMC13139229; doi:10.1007/s43032-026-02069-9)
Supplement: Supplementary file 1 — Supplementary file1 (DOCX 17.6 KB) [file 43032_2026_2069_MOESM1_ESM.docx]

**Supplementary Table 1. Primer sequences for qRT-PCR**

|  | Primer sequences |
| --- | --- |
| *18S* | F:5’-GTAACCCGTTGAACCCCATT-3’ |
|  | R:5’-CCATCCAATCGGTAGTAGCG-3’ |
| *FGF17* | F:5’-AAGACTGCGTGTTCACGGAG-3’ |
|  | R:5’-CAAACTCGAACTGCTTCTGCT-3’ |
| *AKT3* | F:5’-TGAAGTGGCACACACTCTAACT-3’ |
|  | R:5’-CCGCTCTCTCGACAAATGGA-3’ |
| *IRS4* | F:5’-CGACCGCACTCATTGGGAC-3’ |
|  | R:5’-CGCGTTTGCAGACTTCCTC-3’ |
